# Effects of Tetracycline on *Scenedesmus obliquus* Microalgae Photosynthetic Processes

**DOI:** 10.3390/ijms231810544

**Published:** 2022-09-11

**Authors:** Zhehua Chen, Gan Gu, Ziyun Wang, Dong Ou, Xianrui Liang, Changwei Hu, Xi Li

**Affiliations:** 1 College of Biological, Chemical Science and Engineering, Jiaxing University, Jiaxing 314001, China; 2 College of Pharmacy, Zhejiang University of Technology, Hangzhou 310014, China

**Keywords:** tetracycline, photosynthesis, transcriptomics, photosynthesis-related genes, hormesis

## Abstract

Tetracycline (TC) antibiotics can be detected worldwide in the aquatic environment due to their extensive use and low utilization efficiency, and they may affect the physiological processes of non-target organisms. In this study, the acute and sub-acute toxicities of TC on the freshwater microalga *Scenedesmus obliquus* were investigated with an emphasis on algal photosynthesis and transcription alterations during an 8 d TC exposure. The results showed that the IC_10_, IC_30_ and IC_50_ values were 1.8, 4.1 and 6.9 mg/L, respectively. During sub-acute exposure, the microalgae of the IC_10_ treatment was able to recover comparable growth to that of the control by day 7, while significantly lower cell densities were observed in the IC_30_ and IC_50_ treatments at the end of the exposure. The photosynthetic efficiency *F*_v_/*F*_M_ of *S. obliquus* first decreased as the TC concentration increased and then returned to a level close to that of the control on day 8, accompanied by an increase in photosynthetic activities, including light harvesting, electron transport and energy dissipation. Transcriptomic analysis of the IC_10_ treatment (1.8 mg/L TC) revealed that 2157 differentially expressed genes were up-regulated and 1629 were down-regulated compared with the control. KEGG and GO enrichments demonstrated that 28 photosynthesis-related genes involving light-harvesting chlorophyll protein complex, photosystem I, photosystem II, photosynthetic electron transport and enzymes were up-regulated, which may be the factor responsible for the enhanced photosynthesis and recovery of the microalgae. Our work may be helpful not only for gaining a better understanding of the environmental risk of TC at concentrations close to the real levels in natural waters, but also for explaining photosynthesis and related gene transcription induced by antibiotics.

## 1. Introduction

Due to their broad spectrum of activity and low cost, tetracycline (TC) antibiotics are widely used in veterinary and human therapeutic applications [1], and as growth promoters in animal husbandry and aquaculture [2]. Hence, TC production was reported to rank topmost in the world [3]. However, it has been estimated that up to 75% of TCs used by humans and livestock enter the environment because of their low efficiency in metabolism and utilization [4,5]. Moreover, the half-life of TCs in the aquatic environment is relatively long (34–329 h) [6]. Therefore, TCs are frequently detected in natural waters and sediments [7,8], and their concentration varies greatly. For example, the average TC concentration detected in the Yellow Sea of China was 1.55 ng/L [9], while that detected in the Wenyu River in Beijing was as high as 9.5 μg/L [10]. Relatively high TC concentrations (~110 μg/L) were also detected in river samples from Brazil [11]. In some surface waters that receive effluent from wastewater treatment plants, detected TC concentrations have been shown to vary from 3.3 ng/L to 2.0 mg/L [12].

The toxicity of TC toward aquatic organisms has attracted extensive attention, but the current understanding on TC toxicity is still limited [13]. TC in the aquatic environment has been demonstrated to have adverse effects on aquatic organisms, such as crustaceans and fish and especially microalgae, which are unicellular organisms that are sensitive to antibiotics [14]. Halling-Sørensen compared TC inhibition on the growth of the microalga *Selenastrum capricornutum* and the cyanobacterium *Microcystis aeruginosa* [15]. The results showed that *M. aeruginosa* was generally much more sensitive to TC than *S. capricornutum*, with obtained IC_50_ values of 0.09 and 2.2 mg/L, respectively. Xu et al. reported acute toxicity of TC to the microalga *Chlorella vulgaris*, with an obtained 96 h IC_50_ of 7.73 mg/L [16]. It was found that TC exposure resulted in the production of a stress hormone, abscisic acid, in *Lemna minor* [17]. TC could affect the reproductive performance of the large flea *Daphnia magna* [18]. Exposure to TC led to antioxidant effects, neurotoxicity and histological alteration in the freshwater fish *Gambusia holbrooki* [19], and endocrine disruption in male medaka [20].

The toxicological mechanisms of TC on microalgae are not fully understood. TC exposure could change algal structure [16], induce oxidative stress [21,22], interfere with the protein synthesis [21] and influence photosynthesis of the microalgae [23,24]. Because photosynthesis is the most important physiological process for autotrophic microalgae to fix carbon, several studies have attempted to explore the toxicological mechanisms of TC on microalgae in relation to photosynthetic activity. It was reported that the photosynthetic activity (*F*_v_/*F*_M_) of two microalgae, *Dictyosphaerium pulchellum* and *Micractinium pusillum*, was significantly inhibited during an 11 day exposure to 5 mg/L TC [24]. Yang et al. reported that the *F*_v_/*F*_M_ of *M. aeruginosa* decreased sharply at high TC concentrations (0.5–5.0 mg/L), but recovered after 24 h [25]. Some antibiotics are regarded as inhibitors of photosynthesis because they can block electron transport in photosystem II (PSII) [23]. However, the mechanisms of TC on the microalgal photosynthesis process have not been investigated in detail. Thus, studying the photosynthetic activity and photosynthesis-related genes of microalgae could provide a more comprehensive understanding of the effect of TC on microalgae.

Transcriptomics have frequently been used in the fields of biology, ecology and toxicology [26,27]. To date, most studies on TC toxicity have focused on physiological and morphological characteristics, while gene transcription has received less attention. A recent study reported the transcriptomic responses of *M. aeruginosa* to three mixed antibiotics, including TC, and found that 45 up-regulated genes involving ribosome and photosynthesis were significantly (*p* < 0.05) enriched, indicating that *M. aeruginosa* adapted to the antibiotics [2]. Previous research also found that antibiotics could inhibit chloroplast metabolism of the green microalga *Microcystis flos-aquae*, thereby affecting algal growth [28]. However, the effects and underlying mechanisms of TC toward microalgae were not investigated in detail.

Here, we hypothesize that TC may influence photosynthesis and the related gene transcription of the microalga *Scenedesmus obliquus*. The objectives of the present study were to investigate the effects of TC on *S. obliquus* photosynthesis, and to compare the photosynthetic activity and transcriptional characteristics of the microalgae between the TC-treated group with a relatively low TC concentration and the control. Our work helps to better understand the mechanisms of TC on microalgal photosynthesis and the expression of photosynthesis-related genes.

## 2. Materials and Methods

### 2.1. Chemicals and Organism

Tetracycline (CAS No. 60-54-8) was purchased from Shanghai Macklin Inc. (Shanghai, China). The microalga *S. obliquus* (FACHB-417) was purchased from the Freshwater Algae Culture Collection at the Institute of Hydrobiology, China. *Scenedesmus obliquus* was cultured in 500 mL conical flasks containing 300 mL BG11 medium. The culture conditions were maintained at a light intensity of 90 ± 5 μmol/m^2^·s, a light–dark cycle of 12 h:12 h and temperature of 25 ± 0.5 °C. The flasks were hand-shaken at three regular times each day. Fifty percent of the culture was discarded, and an equal volume of fresh medium was added each week to maintain vigorous vitality of *S. obliquus*.

### 2.2. Acute Toxicity Assay

Acute toxicity of TC on *S. obliquus* was investigated according to the standard OECD Guideline 201 [29] with minor modification. The algal culture was diluted to a cell density of 2 × 10^5^ cells/mL with BG 11 medium, and then separated into 250 mL flasks containing 150 mL of culture solution. The TC stock solution prepared with BG11 medium was added to the flasks to achieve starting concentrations of 0, 1, 2, 4, 8, 16 and 32 mg/L. The culture conditions were similar to those mentioned above and five parallel replicates were performed. The exposure period was 96 h. The flasks were hand shaken at three regular times daily. The optical density (wavelength 680 nm) of the culture was determined every 24 h using a spectrophotometer (Rayleigh Analytical Instrument Corp., Beijing, China), and the cell number (×10^6^ cells/mL) *y* was calculated using a growth standard curve (*y* = 9.3946*x*, *R*^2^ = 0.9995) supplemented by cell counting using a hemocytometer. The specific growth rate was used to evaluate the growth and was calculated according to OECD Guidelines [29]. The inhibition concentrations (ICs) of TC on the microalgae (i.e., IC_1_ to IC_100_) were calculated using the plugin “Probit” of the software SPSS 17.0 for Windows.

### 2.3. Exposure Procedure of Sub-Acute Toxicity

Three IC values, specifically IC_10_, IC_30_ and IC_50_, were selected and used to test the sub-acute toxicity. First, 250 mL flasks containing 150 mL algal culture with an initial cell density of 2 × 10^6^ cells/mL were prepared. The TC stock solution was added to the flasks to obtain the three desired TC concentrations. A control group without added TC was also performed. Each group had three replicates. The culture conditions, shaking and cell density monitoring followed the previously mentioned protocol. The experiment lasted eight days.

### 2.4. Photosynthesis Assay

Chlorophyll *a* fluorescence (OJIP) of *S. obliquus* was measured using the Aquapen system (Photon Systems Instruments, AP-C100, Brno, Czech Republic). The test was performed daily at 9:00 am according to [30]. To reflect the photosynthetic performance of the microalgae, the obtained OJIP curves were used, along with eight selected OJIP parameters, specifically *F*_v_/*F*_M_ (the maximum quantum yield of primary photochemistry), *M*_O_ (the slope at the origin of the relative variable fluorescence), *S*_m_ (the working integral of the energy needed to close all reaction centers), *n* (the number of Q_A_ reduction events between time 0 to *t*_P_), ABS/RC (the absorption flux per reaction center (RC)), TR_O_/RC (the trapped energy flux per RC), ET_O_/RC (the electron transport flux per RC) and DI_O_/RC (the dissipated energy flux per RC) [31,32,33,34]. 

### 2.5. Transcriptomics Assay

Total RNA was extracted from the triplicate IC_10_ (1.8 mg/L TC) samples and the control samples after 8 days of growth using Trizol Reagent (Shanghai, China). The NanoDrop 2000 spectrophotometer was employed to determine the concentration and purity of the total RNA of the different samples. The total RNA samples were then submitted to Shanghai Bioprofile Co., Ltd. (Shanghai, China) for preparation and construction of the mRNA library, followed by transcriptomic sequencing on the HiSeq X Ten System (Illumina, San Diego, CA, USA). Sequencing data generally contained a number of connectors and low-quality reads. The Cutadapt (v2.7) software was used to filter the sequencing data to obtain high quality sequence (Clean Data) for further analysis. The clean reads were assembled into contigs and transcripted with Trinity software (http://trinityrnaseq.sf.net; 9 September 2021), and then achieved unigenes by NCBI Blast software (http://www.ncbi.nlm.nih.gov/; 9 September 2021). A more than 2-fold change differential and *p*-value of <0.05 were used to identify the significance of differential expressed genes between two groups. The annotation analysis was conducted according to the previous study [35].

### 2.6. Statistical Analysis

Results are presented as the arithmetic mean with the corresponding standard deviation (*n* = 5 for acute toxicity, *n* = 3 for sub-acute toxicity). Statistical significance of differences observed among treatments was determined via one-way analysis of variance (ANOVA) and covariance (ANCOVA), followed by Tukey’s pair-wise comparison at a significance level of *p* < 0.05.

## 3. Results

### 3.1. Acute Toxicity of TC on Scenedesmus obliquus

The cell density of *S. obliquus* was monitored daily during the 96 h acute exposure, and the inhibition TC concentrations were calculated based on the specific growth rate (Table S1). The estimated values of IC_10_, IC_30_ and IC_50_ were 1.8, 4.1 and 6.9 mg/L, respectively (Table 1). These values were employed for the sub-acute toxicity test in the follow-up experiment.

### 3.2. Algal Growth during the 8 d Sub-Acute Exposure

**Figure 1:**
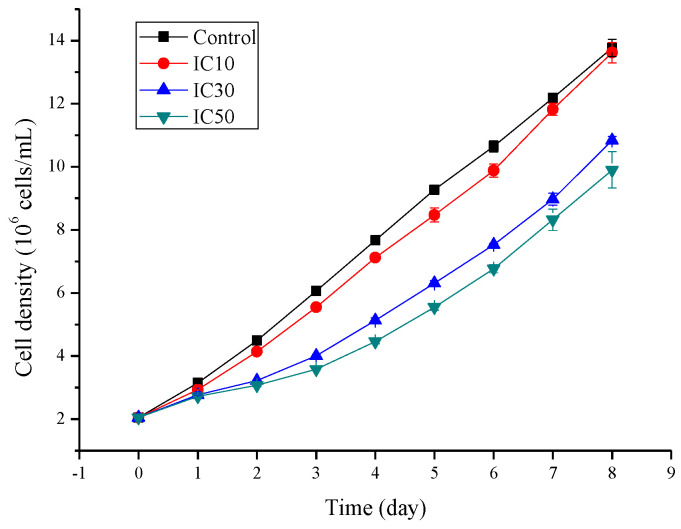
Growth curves of *Scenedesmus obliquus* exposed to different concentrations of tetracycline for 8 days.

The variation in the *S. obliquus* cell density during the 8 d sub-acute exposure is shown in Figure 1. When the microalgae were exposed to TC at the IC_10_ value (1.8 mg/L), the growth was significantly inhibited (*p* < 0.05) compared with that of the control after 2 d exposure, and the inhibition lasted 6 days. However, the cell density of the microalgae in the IC_10_ treatment recovered to the level of that of the control on day 7. For the IC_30_ and IC_50_ treatments, the microalgae suffered more suppression from TC than that of IC_10_ treatment from day 2, and exhibited significantly lower cell densities (*p* < 0.05) at the end of the exposure (day 8).

### 3.3. Photosynthetic Performance of Scenedesmus obliquus Exposed to TC

**Figure 2:**
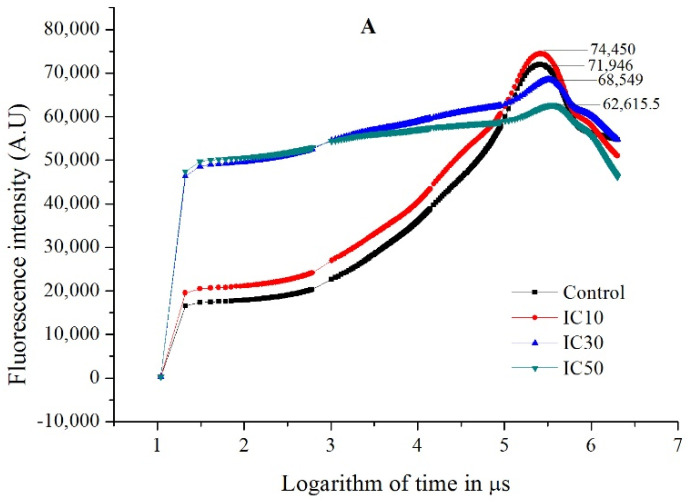

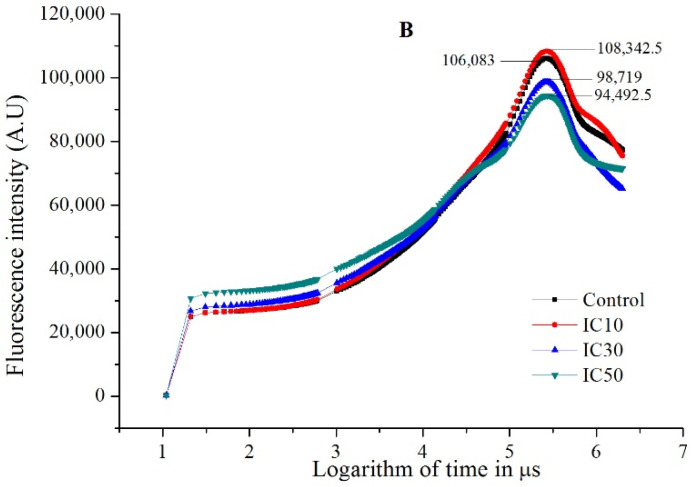
OJIP curves of *Scenedesmus obliquus* after exposure to different concentrations of tetracycline for 4 days (**A**) and 8 days (**B**).

For the OJIP curves, the initial fluorescence is defined as O (also known as the *F*_o_ value at 20 μs). J and I represent intermediate values at the times of ~2 to 3 ms and ~30 ms, respectively. P, also known as the *F*_m_ value, is the peak value in the OJIP curve. After TC exposure in the IC_10_ treatment for 4 days, the OJIP curve of *S. obliquus* started with a significantly higher (*p* < 0.05) O level than the control, and then the fluorescence remained high and achieved a higher *p* value than the control (Figure 2A). For the IC_30_ and IC_50_ treatments, much higher O values were observed (approximately 2.8 times of the control). The delayed P (*F*_M_) values were significantly lower (*p* < 0.05) than the control. The OJIP curves of both treatments appeared flat, without remarkable J and I values.

After the 8 d exposure, the OJIP curves of all three TC treatments (IC_10_, IC_30_ and IC_50_) basically followed those of the control in the O-I phase (Figure 2B). However, relatively high TC concentrations (IC_30_ and IC_50_) resulted in significantly lower (*p* < 0.05) *p* values than the control, while the IC_10_ treatment achieved a *p* value close to the control.

The eight selected photosynthetic parameters were compared among the four groups, and the results are listed in Table 2. After the 4 d exposure, TC ranging from 1.8 to 6.9 mg/L exhibited a distinct dose–response relationship on the microalga *S. obliquus*. As the TC concentration increased, the *F*_v_/*F*_m_ (the maximum quantum yield of the primary photochemistry) decreased significantly (*p* < 0.05), with the *F*_v_/*F*_m_ values of the IC_10_, IC_30_ and IC_50_ treatments dropping to 95%, 42% and 30%, respectively, of those of the control. However, the other seven parameters all increased, with several parameters increasing to high levels. For example, relative to the control, for the IC_10_, IC_30_ and IC_50_ treatments, the *M*_O_ increased by 1.2, 3.4 and 4.3 times, respectively; *n* increased by 1.2, 5.2 and 8.9 times, respectively; and ABS/RC increased by 1.1, 3.9 and 5.9 times, respectively. TR_O_/RC (trapped energy flux per RC) and ET_O_/RC were less affected than the other parameters, with the maximum magnitudes of increase in IC_50_ treatment being 1.8 and 1.3 times, respectively. The biggest change was observed for DI_O_/RC, which was 18.3 times that of the control in the IC_50_ treatment. It could be seen that exposure to TC with the IC_10_ value caused a minimum influence on the photosynthesis of the microalga *S. obliquus*.

After the 8 d exposure, however, the stress of TC on the photosynthetic performance was evidently alleviated. For the IC_10_ treatment, all parameters were close to those of the control and showed no significant difference except for *F*_v_/*F*_m_, which was significantly lower (*p* < 0.05) than that of the control. Most of parameters of the IC_30_ and IC_50_ treatments were still different from those of the control, but the differences had lessened compared with those of the 4 d exposure. 

### 3.4. Transcriptional Responses of Scenedesmus obliquus Exposed to TC

**Figure 3:**
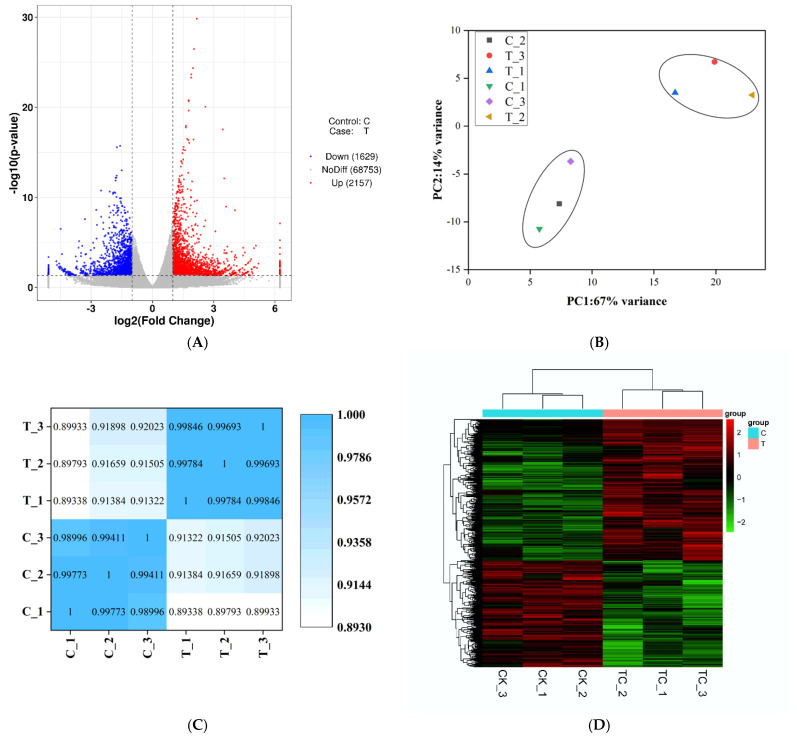
Transcriptomic profiling of *Scenedesmus obliquus* in the control (C) and tetracycline treatment (T) after 8 d exposure. (**A**) Volcano plot based on the differentially expressed genes (DEGs) in T vs. C groups; (**B**) principal components analysis based on the expression quantity; (**C**) correlation analysis of patterns of gene expression; (**D**) a heatmap of the centered and scaled FPKM values of DEGs.

RNA-seq transcriptomic analysis was performed for the 1.8 mg/L TC (IC_10_) treatment and the control at the end of the exposure. A total of 72,539 transcripts were detected in *S. obliquus*. Compared with the control, 3786 transcripts were identified as differentially expressed genes (DEGs; *p* < 0.05 and |log_2_FoldChange| ≥ 1) in the IC_10_ treatment, of which 2157 represented up-regulated genes and 1629 represented down-regulated genes (Figure 3A). Principal component analysis (PCA) showed that the gene-expression clusters in the IC_10_ treatment and the control differed (Figure 3B). Furthermore, correlation of gene expression among the three replicates of the two groups was significant (Figure 3C), which was also reflected by the heatmap assay (Figure 3D).

**Figure 4:**
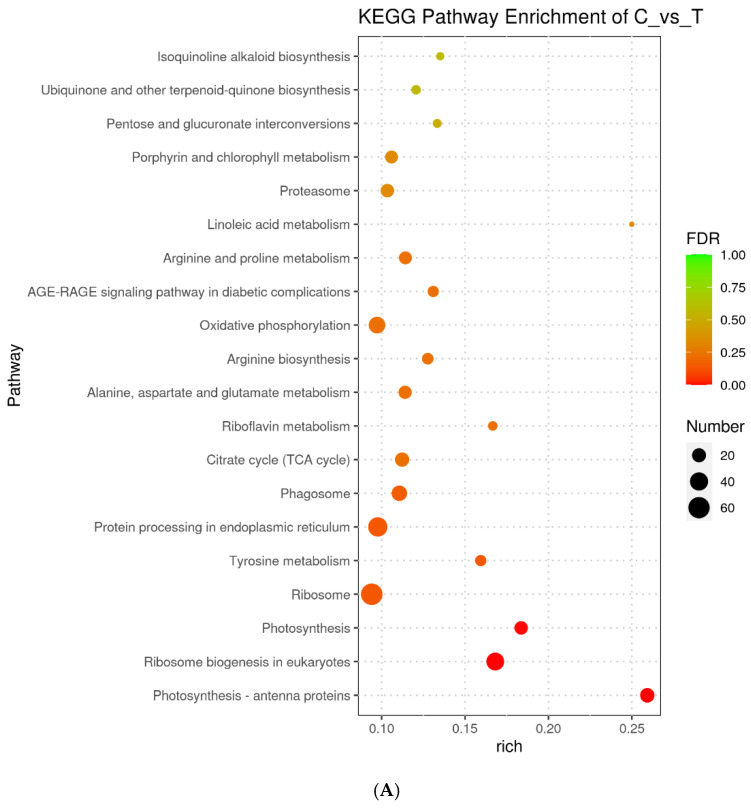

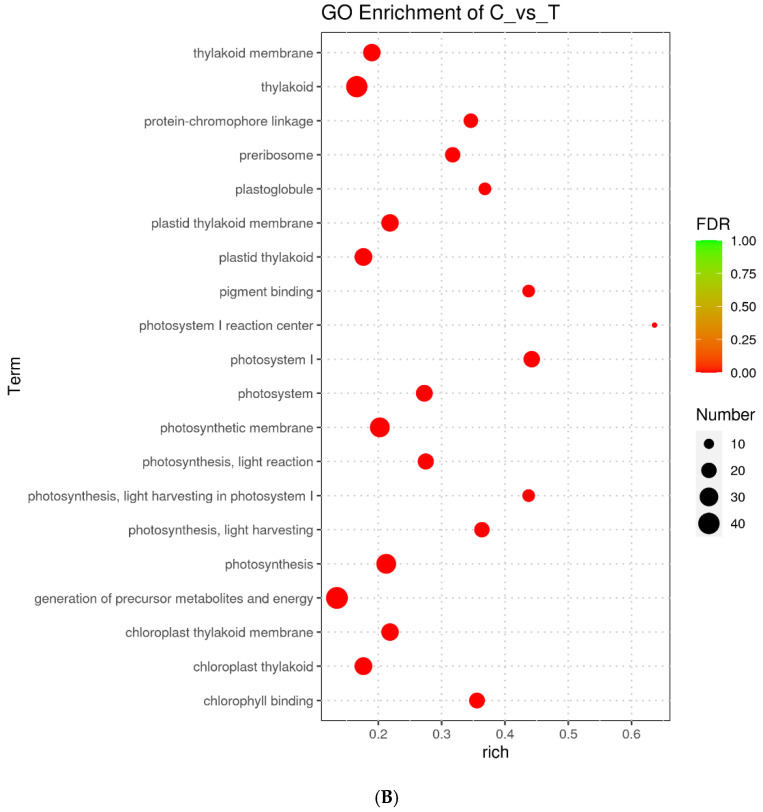
Scatter plots of enrichment results for the KEGG pathways (**A**) and GO function enrichment (**B**) for the IC_10_ treatment of *Scenedesmus obliquus* compared with the control (C). The *x* axis shows the rich factor (the ratio of differentially expressed genes and total gene numbers of the pathway) for each KEGG or GO pathway. The color of the dot indicates the FDR value; the size of the dot denotes the number of genes involved in each pathway.

To investigate the molecular mechanisms of microalgal performance after TC treatment, changes in the expression of specific pathways were investigated and compared between the IC_10_ treatment and the control. First, the metabolic pathways were analyzed using KEGG Orthology (KO), and the top 20 enriched pathways are shown in Figure 4A and Table S2. Photosynthesis–antenna proteins, photosynthesis and ribosome biogenesis in eukaryotes were identified as the most significantly enriched pathways. The former two pathways were involved in photosynthesis, in which 38 DEGs were up-regulated and only one DEG was down-regulated. As for the ribosome biogenesis pathway, four DEGs were up-regulated and 34 DEGs were down-regulated. Among the 20 pathways, the other 17 pathways were related to ribosome, amino acid biosynthesis, citrate cycle, oxidative phosphorylation, chlorophyll metabolism, quinone biosynthesis, etc. Next, the Goseq tool was used to perform Gene Ontology (GO) enrichment analysis, and the top 20 enriched pathways are shown in Figure 4B and Table S3. It is worth noting that the DEGs in all the 20 pathways were up-regulated, except that 17 of the 20 DEGs in the preribosome pathway were down-regulated. Seven pathways, specifically photosystem I (PSI) (GO:0009522), photosynthesis-light harvesting (GO:0009765), photosynthesis (GO:0015979), photosystem (GO:0009521), photosynthesis-light reaction (GO:0019684), photosynthesis-light harvesting in PSI (GO:0009768) and PSI reaction center (GO:0009538), were involved in photosynthesis. Additionally, nine pathways, specifically photosynthetic membrane (GO:0034357), thylakoid (GO:0009579), chloroplast thylakoid membrane (GO:0009535), plastid thylakoid membrane (GO:0055035), thylakoid membrane (GO:0042651), chloroplast thylakoid (GO:0009534), plastid thylakoid (GO:0031976), chlorophyll binding (GO:0016168) and pigment binding (GO:0031409), were related to the chloroplast structure or pigment binding.

**Figure 5:**
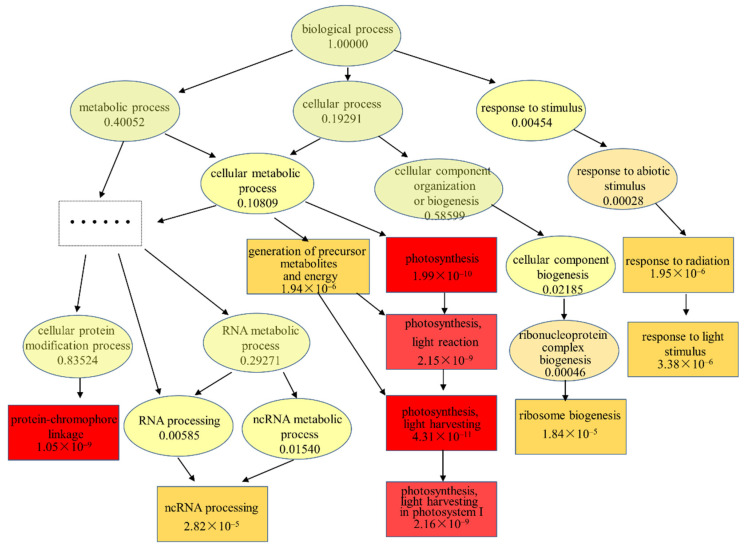
Significantly enriched Gene Ontology (GO) biological process terms and up-regulated differentially expressed genes (DEGs) in *Scenedesmus obliquus* exposed to tetracycline, compared to the control. The colors of the nodes denote the *p*-value (decreasing with the deepening of color from light yellow to deep red), which is also shown after each item. The numbers in each term represent DEGs and the total genes involved in this term. The top 10 GO terms are displayed in the rectangular frames.

The results of the GO enrichment, in terms of biological processes, are presented in Figure 5 based on the hierarchical relationship of the GO terms. The node color denotes the *p*-value, which decreased in the following order: of light green, yellow, orange and red. The top 10 GO terms are displayed in rectangular frames. It can be seen that light harvesting of the light reaction in PSI during photosynthesis was significantly up-regulated (14 DEGs in a total 32 of genes, *p*-value 2.2 × 10^−9^). The illustration of cellular components of the GO enrichment is shown in Figure S1A. The genes related to PSI and the photosynthetic membrane were significantly up-regulated and enriched. As for the molecular function, the genes that regulate pigment binding and chlorophyll binding were most significantly enriched.

**Figure 6:**
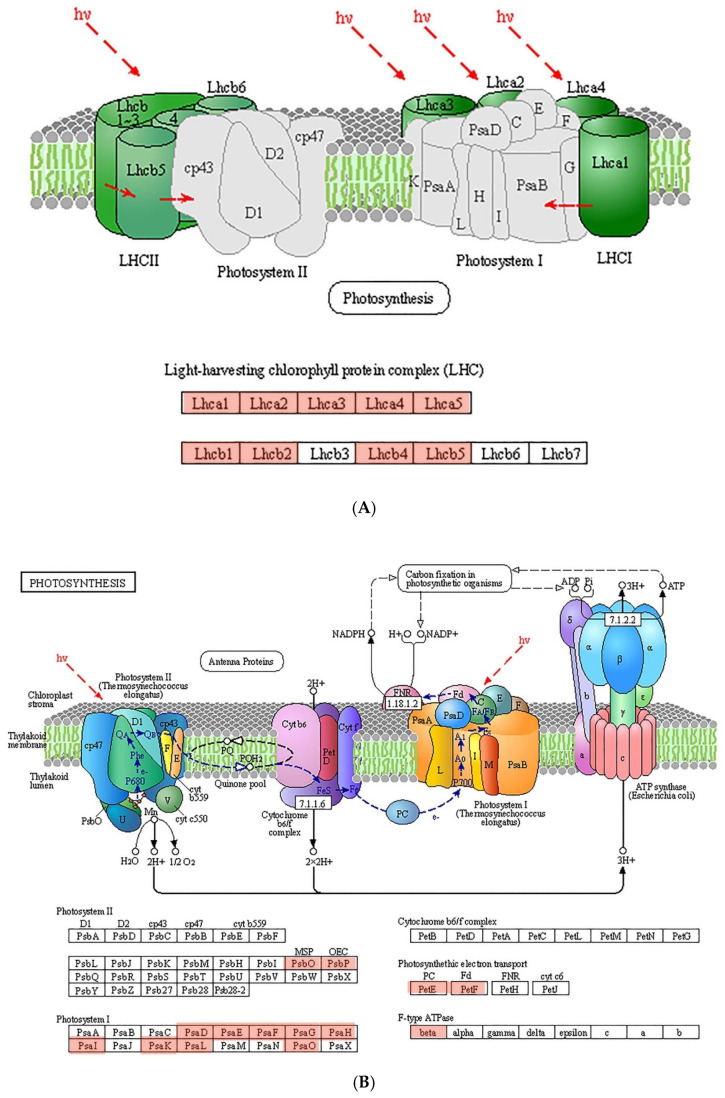
Schematic of the light-harvesting chlorophyll protein complex (**A**) and photosystem (**B**). Proteins and subunits are distinguished by different colors. The up-regulated proteins are marked in light red at the bottom of figure.

To reveal the relationship of photosynthesis-related genes and proteins, all genes involving photosynthesis, photosynthetic pigments and enzymes in both the KEGG and GO enrichments were summarized, and their functions were described based on KEGG Orthology (KO). A total of 41 genes were extracted, and genes with the same function were simplified as a single gene, maintaining the one with the lowest *p*-value. The subsequently obtained 28 genes with their KO entry, fold change, *p*-value and description are listed in Table S4. Overall, these genes were related to the light-harvesting chlorophyll protein complex (LHC), PSI, photosynthetic electron transport and enzymes. LHCI and LHCII contain five and seven subunits, respectively. All of the genes related to LHCI (*Lhca*1–5) were up-regulated, while four of seven genes related to LHCII were up-regulated (Figure 6A). Nine genes and two genes were up-regulated in PSI and PSII, respectively (Figure 6B). As for others, two genes (*petE* and *petF*) were related to photosynthetic electron transport, one gene (*atpD*) was related to ATPase, and three genes were related to enzymes of chlorophyll. 

## 4. Discussion

### 4.1. Toxicity of Tetracycline on Microalgae

Antibiotics were originally designed to act against pathogenic bacteria, but their impact on non-target organisms, such as microalgae, has also been documented [36,37]. Microalgae are primary producers; thus, a change in their population may tremendously affect the health of the ecosystem. Furthermore, it was found that several bacterial receptors and/or pathways are conserved in microalgae [38,39]. Therefore, it is meaningful to explore the toxicity and underlying mechanisms of antibiotics on microalgae.

Microalgae have been demonstrated as being highly sensitive to TC, compared to zebrafish embryos, fish cells and *Vibrio fischeri* [40]. A limited number of studies have reported the IC_50_ value of TC for microalgae ranging from 2–8 mg/L. For example, the 72 h IC_50_ of TC for the green microalga *S. capricornutum* was reported as 2.2 mg/L [15]. In our study, the 96 h IC_50_ of TC for *S. obliquus* was 6.9 mg/L, which was close to the 96 h IC_50_ of 7.73 mg/L for the microalga *C. vulgaris* reported by Xu et al. [16]. The cyanobacterium *M. aeruginosa* showed a more sensitive response to TC stress, with a 168 h IC_50_ of 0.09 mg/L reported by Halling-Sørensen [15], perhaps due to their bacterial nature.

Although the effects of antibiotics on microalgae are possibly acute, inhibition of algal growth by antibiotics does not always occur because of relatively low antibiotic concentrations as detected in natural waters, ranging from ng/L in groundwater to mg/L in hospital effluent [41]. Depending on their concentration, antibiotics may exert biphasic biological effects on organisms. This phenomenon, also known as hormesis or “low-dose stimulation and high-dose inhibition” [42], has been reported for microalgae to a limited extent [22,43,44]. It was found that low concentrations of chlortetracycline hydrochloride (2 and 5 mg/L) could promote microcystin-LR production in *M. aeruginosa* [45]. Growth of the microalga *Coelastrella* sp. was promoted by 0.5–2.0 mg/L TC [22], while *Synechocystis* sp. could be stimulated by an extremely low dose of TC (0.1 μg/L) [46]. Therefore, investigating the response of microalgae to low dosages of TC may provide more information and may be of practical significance for understanding the environmental risk of this antibiotic.

### 4.2. Effects of Tetracycline on Algal Photosynthesis Performance

The mechanisms of action of antibiotics on microalgae mainly include three aspects, i.e., photosynthesis, oxidative stress and macromolecular alterations [47]. For plants and microalgae, the most important and crucial biological process is photosynthesis, which fixes carbon for energy metabolism and other physiological processes. Hence, a plethora of previous research has used photosynthetic efficiency as an important index to evaluate antibiotic toxicity. 

The photosynthetic parameter *F*_v_/*F*_M_ is frequently used to assess photosynthetic efficiency. Exposure to 5 mg/L TC could inhibit the *F*_v_/*F*_M_ of the microalgae *D. pulchellum* and *M. pusillum* [24]. In contrast to a high dosage, a low dosage of TC (0.05 mg/L) was found to enhance the *F*_v_/*F*_M_ of the macroalga *M. aeruginosa*, which also exhibited resilience under exposure to higher dosages (0.5, 1.0, 2.0 and 5.0 mg/L) [25]. Our results demonstrate that the *F*_v_/*F*_M_ of *S. obliquus* first decreased with exposure to TC (1.8, 4.1 and 6.9 mg/L) and then recovered to a level close to that of the control on day 8 (Table 2), which was basically consistent with the finding of Yang et al. [25].

The detailed photosynthetic process and parameters, which could provide more information on photosynthesis, have not received enough attention in previous research. In our study, variations in other selected photosynthetic parameters were quite different from *F*_v_/*F*_M_ (Figure 2, Table 2). On the contrary, these parameters all increased with the increasing TC concentration. With TC exposure ranging from 1.8 to 6.9 mg/L, the absorption flux and trapped energy flux per RC increased, depending on the TC dosage, indicating that the microalga *S. obliquus* attempted to promote light harvesting and use of absorbed light. *S*_m_ reflects single-turnover *Q*_A_ reduction events; *M*o reflects the maximum rate of *Q*_A_ reduction during the O-J process [48]; and *n* expresses the frequency of *Q*_A_ reduction events during the fluorescence transient interval from t_0_ to t_P_ [33]. The increase in these parameters after a 4 d exposure to TC indicated the increase in electron transport, which was demonstrated by the increase in ET_O_/RC. *Q*_A_ was the secondary plastoquinone acceptor in the PSII electron transport pathway (P_680_→pheophytin→*Q*_A_→*Q*_B_) [49]. The reduction rate of *Q*_A_ was a key event for electron transfer [50]. The dissipated energy flux per RC (i.e., DI_O_/RC) also increased with the increasing TC concentration, and exhibited the largest amplification among all 8 parameters, reaching an extremely high level (18.3 times that of the control). The increase in DI_O_/RC means more energy dissipation, which may be a disadvantage for microalgae in maintaining their photosynthesis. This may be responsible for the low photosynthetic efficiency (*F*_v_/*F*_M_), and could also be a protection strategy of microalgae against excessive absorption of light energy [51].

In the present study, although the growth and photosynthesis of *S. obliquus* in the TC treatments did not exceed the control, the increased light use and electron transport may be regarded as being partially due to a hormesis effect induced by TC in all three treatments. Under exposure to the lowest TC (1.8 mg/L), the microalgae achieved a significantly higher *F*_M_ (Figure 2A) on day 4 and a similar cell density on day 8 compared to the control, suggesting a hormesis effect caused by 1.8 mg/L TC.

### 4.3. Effects of Tetracycline on Photosynthesis-Related Genes

Some antibiotics have been demonstrated to influence algal photosynthesis and macromolecular metabolism, the mechanisms of which include disturbing the transcription of certain genes related to protein synthesis, pigment synthesis and enzyme activity [52,53]. Thus, the transcription of *S. obliquus* exposed to TC could cause molecular changes that are responsible for the TC effects related to growth and photosynthesis.

Microalgae possess an oxygenic photosynthesis system comprising PSI, PSII, LHCs and electron carriers that are localized in the thylakoid membranes [54]. PSI is a protein complex comprising 15 core subunits (PsaA to PsaL, and PsaN to PsaP) that mediate light-driven electron transport from water to NADPH [55]. PSII, which is regarded as the heart of photosynthesis, mediates the production of high-energy electrons and oxygen from water [56]. According to previous studies, PSII is more sensitive than PSI to various environmental stressors, including antibiotics [57,58]. However, our study shows that more proteins were affected in PSI than PSII (Figure 6B). The up-regulated proteins of PSI included PsaD to PsaJ, PsaL and PsaO, which have multiple functions, including binding of ferredoxin, PSI subunits, LHCs and electron transport, and stabilization of PSI subunits [55]. Two genes (*psbO* and *psbP*) related to PSII proteins were up-regulated in our study. The manganese stabilizing protein (MSP, encoded by *psbO*) and the oxygen evolving center (OEC, encoded by *psbP*) are essential for maintaining the maximum rates of oxygen evolution and water oxidation during photosynthesis [59].

LHCs are important pigment-binding components of the thylakoid membrane and serve as coordinators of antenna pigments [60]. Plastocyanin and ferredoxin are important components of the photosynthetic electron transport chain, and they determine the capacity of forward electron transport towards carbon assimilation [61]. In our study, most of the LHC-related genes and the genes encoding plastocyanin and ferredoxin were up-regulated, which may explain the increased light harvesting and electron transport illustrated by the photosynthetic parameters and the recovery of *S. obliquus* under exposure to 1.8 mg/L TC.

The up-regulation of photosynthesis-related proteins induced by TC in microalgae and cyanobacteria has also been reported in two previous studies. Cui et al. (2020) found that 100 ng/L of TC enhanced the photosynthetic activity of the microalga *Synechocystis* sp. PCC 6803 by up-regulating photosynthesis-related proteins, including PsaF, PetB, PetD and PsbF. Xu et al. (2022) reported that 150 ng/L of mixed antibiotics (TC, ciprofloxacin and sulfamethoxazole) resulted in the up-regulation of eight photosynthesis-related genes, including *petE*, *psaD* and *psaF*, in *M. aeruginosa*, and three photosynthesis-related genes, including *petB* and *psaK2*, in *Synechocystis* sp.

Since the antibiotic concentration is usually much lower than the IC_50_ values obtained in toxicological research, the hormesis effect of low-concentration antibiotics on microalgae may frequently occur in natural waters [47]. Thus, this phenomenon deserves greater research and public concern to acquire a comprehensive understanding of the environmental risk of antibiotics. In addition, the transfer and bioaccumulation of TC in the food chain and generation of resistance genes in organisms should not be negligible.

## 5. Conclusions

In this study, the acute toxicity test of TC on the freshwater microalga *Scenedesmus obliquus* was performed, and the values of IC_10_, IC_30_ and IC_50_ of TC for the microalga *S. obliquus*, which were obtained from 96 h acute toxicity assays, were used for sub-acute toxicity evaluation. The variation in algal photosynthetic activity under the three TC concentrations and the transcriptional responses under 1.8 mg/L TC exposure were investigated. The cell density of the microalgae in the IC_10_ treatment recovered to the level of that of the control on day 7, while *S. obliquus* in the IC_30_ and IC_50_ treatments achieved significantly lower cell densities at the end of exposure. The *F*_v_/*F*_M_ of *S. obliquus* decreased with exposure to TC, and then recovered close to level of that of the control in day 8. TC exposure resulted in promotion of light use and electron transport, which increased depending on TC concentration (1.8 to 6.9 mg/L). The frequency of *Q*_A_ reduction was also facilitated by TC. However, the photosynthetic efficiency of the microalgae decreased due to more energy dissipation. The results of the transcriptomics assay revealed that nine DEGs of PSI, two DEGs of PSII and nine DEGs of LHC were up-regulated after 8 d exposure to 1.8 mg/L of TC. Evidence from KEGG and GO enrichment demonstrated up-regulation of photosynthesis-related genes involving LHC, PSI, PSII, photosynthetic electron transport and enzymes, which may account for the promotion of photosynthesis and recovery of the microalgae. Our work may be beneficial for comprehensively understanding the environmental impact of TC in the aquatic environment.

## Figures and Tables

**Table 1:** Estimated tetracycline (TC) concentrations impacting *Scenedesmus obliquus* after 96 h of acute exposure.

Probability	95% Confidence Limits for TC Concentration (mg/L)		
	Estimate	Lower Bound	Upper Bound
**IC_10_**	1.8	0.5	3.1
**IC_30_**	4.1	2.1	6.2
**IC_50_**	6.9	4.3	11.1

**Table 2:** Comparison of the photosynthetic parameters of the microalga *Scenedesmus obliquus* exposed to tetracycline for 4 and 8 days in the different treatments. For each parameter, the data with the same superscript letter indicate no significant difference (*p* < 0.05).

Time (day)	Parameters	Treatment			
		Control	IC_10_	IC_30_	IC_50_
**4**	*F*_v_/*F*_m_	0.76 ± 0.00 ^a^	0.72 ± 0.00 ^b^	0.32 ± 0.02 ^c^	0.23 ± 0.00 ^d^
	*M*_o_	0.13 ± 0.01 ^a^	0.16 ± 0.00 ^b^	0.44 ± 0.01 ^c^	0.56 ± 0.04 ^d^
	*S*_m_	307.0 ± 7.3 ^a^	332.0 ± 1.0 ^b^	947.7 ± 68.0 ^c^	1501.1 ± 18.6 ^d^
	*n*	224.9 ± 0.6 ^a^	265.8 ± 11.5 ^b^	1172.4 ± 100.1 ^c^	2005.2 ± 108.6 ^d^
	ABS/RC	0.97 ± 0.03 ^a^	1.11 ± 0.06 ^b^	3.82 ± 0.23 ^c^	5.72 ± 0.21 ^d^
	TRO/RC	0.73 ± 0.02 ^a^	0.80 ± 0.04 ^b^	1.24 ± 0.02 ^c^	1.34 ± 0.06 ^d^
	ETO/RC	0.60 ± 0.01 ^a^	0.64 ± 0.04 ^a^	0.80 ± 0.01 ^b^	0.77 ± 0.05 ^b^
	DIO/RC	0.24 ± 0.01 ^a^	0.31 ± 0.02 ^b^	2.58 ± 0.22 ^c^	4.38 ± 0.15 ^d^
**8**	*F*_v_/*F*_m_	0.75 ± 0.00 ^a^	0.72 ± 0.00 ^b^	0.73 ± 0.01 ^b^	0.65 ± 0.03 ^c^
	*M*_o_	0.14 ± 0.01 ^a^	0.15 ± 0.00 ^a^	0.15 ± 0.01 ^a^	0.19 ± 0.02 ^b^
	*S*_m_	121.9 ± 8.0 ^a^	118.4 ± 0.6 ^a^	363.1 ± 30.8 ^b^	304.8 ± 107.7 ^b^
	*n*	82.1 ± 5.4 ^a^	79.2 ± 0.14 ^a^	264.1 ± 34.0 ^b^	255.6 ± 119.9 ^b^
	ABS/RC	0.93 ± 0.00 ^a^	0.93 ± 0.00 ^a^	1.00 ± 0.05 ^b^	1.31 ± 0.13 ^c^
	TRO/RC	0.67 ± 0.00 ^a^	0.67 ± 0.00 ^a^	0.73 ± 0.03 ^a^	0.85 ± 0.05 ^b^
	ETO/RC	0.53 ± 0.00 ^a^	0.52 ± 0.00 ^a^	0.58 ± 0.03 ^b^	0.66 ± 0.04 ^c^
	DIO/RC	0.25 ± 0.00 ^a^	0.26 ± 0.00 ^a^	0.27 ± 0.02 ^a^	0.46 ± 0.08 ^b^

## Data Availability

The authors confirm that all data in this experiment are available in the main text and Supplementary Information.

## Supplementary Materials

The following supporting information can be downloaded at: https://www.mdpi.com/article/10.3390/ijms231810544/s1.

## Abbreviations

ABS/RC	The absorption flux per reaction center
DEG	Differentially expressed genes
DI_O_/RC	The dissipated energy flux per reaction center
ET_O_/RC	The electron transport flux per reaction center
*F_O_* (O)	Basal (initial) level of chlorophyll *a* fluorescence at 20 μs
*F_M_* (P)	Maximal level of chlorophyll *a* fluorescence
*F*_v_/*F*_M_	The maximum quantum yield of primary photochemistry
GO	Gene Ontology
IC	Inhibition concentration
KO	KEGG Orthology
LHC	The light-harvesting chlorophyll protein complex
*M*_O_	The slope at the origin of the relative variable fluorescence)
*n*	The number of Q_A_ reduction events between time 0 to *t*_P_)
OEC	The oxygen evolving center
PCA	Principal component analysis
PS	Photosystem
*Q*_A_	The first quinone electron acceptor of Photosystem II
*Q*_B_	The second quinone electron acceptor of Photosystem II
RC	Reaction center
*S*_m_	The working integral of the energy needed to close all reaction centers)
TC	Tetracycline
TR_O_/RC	The trapped energy flux per reaction center
