# Supplementary material for: Effects of Tetracycline on Scenedesmus obliquus Microalgae Photosynthetic Processes
Source: Int J Mol Sci. 2022 Sep 11;23(18):10544. doi: 10.3390/ijms231810544 (PMC9504007; doi:10.3390/ijms231810544)
Supplement: Supplementary file 1 [file ijms-23-10544-s001.zip › ijms-1897184-supplementary.pdf]

Tetracycline-induced alterations of photosynthetic process and transcription in the freshwater microalga *Scenedesmus obliquus*

Zhehua Chen[a,b], Gan Gu[a,b], Xianrui Liang[b], Dong Ou[a,*], Changwei Hu[a,*]

[a]*College of Biological, Chemical Science and Engineering, Jiaxing University, Jiaxing 314001, P.R. China*

[b]*College of Pharmacy, Zhejiang University of Technology, Hangzhou 310014, China*

July 12, 2022

Submitted to: *International Journal of Molecular Sciences*

6 pages
4 tables
1 figure

Table S1 The average cell density and specific growth rate of the microalgae *Scenedesmus obliquus* and the inhibition rate of tetracycline on the microalgae in the 96-h acute toxicity test.

Table S2 Top 20 enriched KEGG items of the microalga *Scenedesmus obliquus* after 8-d exposure to 1.8 mg/L tetracycline (IC_{10} treatment).

Table S3 TOP 20 enriched GO items of the microalga *Scenedesmus obliquus* after 8-d exposure to 1.8 mg/L tetracycline (IC_{10} treatment).

Table S4 The list and description of DEGs in the IC_{10} treatment (1.8 mg/L tetracycline) and the control.

Figure S1 Cytoscape visualization of the BiNGO clustering results of significantly enriched Gene Ontology (GO) cellular component (A) and molecular function (B) terms of up-regulated genes in *Scenedesmus obliquus* exposed to TC, compared to the control. The color of the nodes denotes the *p*-value (decrease with the deepening of color from light yellow to deep red). The numbers in each term represent differentially expressed genes (DEGs) and total genes involved in this term. The top 10 GO terms are displayed in rectangular frames.

Table S1 The average cell density and specific growth rate of the microalgae *Scenedesmus obliquus* and the inhibition rate of tetracycline on the microalgae in the 96-h acute toxicity test.

Items	Tetracycline concentration (mg/L)						
	0	1	2	4	8	16	32
Average cell density at day 0 ($\times 10^6$ cell/mL)	0.207	0.207	0.207	0.207	0.207	0.207	0.207
Average cell density at day 4 ($\times 10^6$ cell/mL)	2.010	1.645	1.465	1.136	0.714	0.322	0.208
Specific growth rate	0.569	0.519	0.490	0.426	0.310	0.111	0.000
Inhibition rate (%)	0	8.8	13.9	25.1	45.5	80.5	100.0

Table S2 Top 20 enriched KEGG items of the microalga *Scenedesmus obliquus* after 8-d exposure to 1.8 mg/L tetracycline (IC_{10} treatment).

Pathway ID	Pathway	Up	Down	DEG	Total	*p*-value	FDR
ko00196	Photosynthesis - antenna proteins	21	0	21	81	6.673E-08	7.407E-06
ko03008	Ribosome biogenesis in eukaryotes	4	34	38	226	1.824E-07	1.012E-05
ko00195	Photosynthesis	17	1	18	98	9.492E-05	0.0035
ko03010	Ribosome	55	7	62	659	0.00564	0.1413
ko00350	Tyrosine metabolism	10	1	11	69	0.00652	0.1413
ko04141	Protein processing in endoplasmic reticulum	42	5	47	481	0.00764	0.1413
ko04145	Phagosome	24	2	26	235	0.00968	0.1535
ko00020	Citrate cycle (TCA cycle)	19	2	21	187	0.0161	0.2228
ko00740	Riboflavin metabolism	3	4	7	42	0.0219	0.2244
ko00250	Alanine, aspartate and glutamate metabolism	17	0	17	149	0.0247	0.2244
ko00220	Arginine biosynthesis	12	0	12	94	0.0252	0.2244
ko00190	Oxidative phosphorylation	29	3	32	329	0.0259	0.2244
ko04933	AGE-RAGE signaling pathway in diabetic complications	11	0	11	84	0.0265	0.2244
ko00330	Arginine and proline metabolism	13	3	16	140	0.0283	0.2244
ko00591	Linoleic acid metabolism	3	0	3	12	0.0434	0.3210
ko03050	Proteasome	18	0	18	174	0.0492	0.3341
ko00860	Porphyrin and chlorophyll metabolism	15	1	16	151	0.0512	0.3341
ko00040	Pentose and glucuronate interconversions	4	2	6	45	0.0824	0.5082
ko00130	Ubiquinone and other terpenoid-quinone biosynthesis	5	2	7	58	0.0969	0.5663
ko00950	Isoquinoline alkaloid biosynthesis	4	1	5	37	0.1034	0.5738

Table S3 TOP 20 enriched GO items of the microalga *Scenedesmus obliquus* after 8-d exposure to 1.8 mg/L tetracycline (IC_{10} treatment).

GO.ID	Term	Up	Down	DEG	Total	*p*-value	FDR
GO:0009522	photosystem I	23	0	23	52	2E-14	3.846E-11
GO:0009765	photosynthesis, light harvesting	20	0	20	55	4.3E-11	2.949E-08
GO:0016168	chlorophyll binding	21	0	21	59	4.6E-11	2.949E-08
GO:0015979	photosynthesis	34	0	34	160	2E-10	9.615E-08
GO:0009521	photosystem	24	0	24	88	9.8E-10	3.205E-07
GO:0018298	protein-chromophore linkage	18	0	18	52	1E-09	3.205E-07
GO:0030684	preribosome	3	17	20	63	1.4E-09	3.846E-07
GO:0034357	photosynthetic membrane	34	0	34	168	1.9E-09	4.231E-07
GO:0019684	photosynthesis, light reaction	22	0	22	80	2.2E-09	4.231E-07
GO:0009768	photosynthesis, light harvesting in photosystem I	14	0	14	32	2.2E-09	4.231E-07
GO:0031409	pigment binding	14	0	14	32	3.5E-09	6.119E-07
GO:0009579	thylakoid	40	0	40	241	2.9E-08	4.395E-06
GO:0009535	chloroplast thylakoid membrane	26	0	26	119	3.2E-08	4.395E-06
GO:0055035	plastid thylakoid membrane	26	0	26	119	3.2E-08	4.395E-06
GO:0010287	plastoglobule	14	0	14	38	5E-08	6.410 E-06
GO:0042651	thylakoid membrane	26	0	26	137	6.3E-07	7.572E-05
GO:0009538	photosystem I reaction center	7	0	7	11	1.3E-06	1.471E-04
GO:0009534	chloroplast thylakoid	27	0	27	153	1.7E-06	1.721E-04
GO:0031976	plastid thylakoid	27	0	27	153	1.7E-06	1.721E-04
GO:0006091	generation of precursor metabolites and energy	42	0	42	312	1.9E-06	1.740E-04

Table S4 The list and description of DEGs in the IC_{10} treatment (1.8 mg/L tetracycline) and the control.

Gene name	KO entry	Fold change (T/C)	*p*-value	Description
*psa*D	K02692	2.182	4.783E-10	photosystem I subunit II
*psa*E	K02693	2.660	9.473E-15	photosystem I subunit IV
*psa*F	K02694	2.259	1.101E-10	photosystem I subunit III
*psa*G	K08905	2.349	2.135E-11	photosystem I subunit V
*psa*H	K02695	3.122	1.156E-18	photosystem I subunit VI
*psa*I	K02696	3.404	1.669E-21	photosystem I subunit VIII
*psa*K	K02698	2.531	4.318E-13	photosystem I subunit X
*psa*L	K02699	3.600813112	0.00362	photosystem I subunit XI
*psa*O	K14332	2.468	2.169E-12	photosystem I subunit PsaO
*psb*O	K02716	2.148	9.112E-10	photosystem II oxygen-evolving enhancer protein 1
*psb*P	K02717	2.310	2.523E-11	photosystem II oxygen-evolving enhancer protein 2

*pet*E	K02638	2.033	1.465E-08	plastocyanin
*pet*F	K02639	2.925	0.00272	ferredoxin
ATPF1B, *atp*D	K02112	3.379	0.03272	F-type H+/Na+-transporting ATPase subunit beta
*chl*I, *bch*I	K03405	15.970	0.02888	magnesium chelatase subunit I [EC:6.6.1.1]
*chl*P, *bch*P	K10960	2.175	0.000169	geranylgeranyl diphosphate/ geranylgeranyl-bacteriochlorophyllide a reductase
por	K00218	2.117	0.00271	protochlorophyllide reductase
*Lhca*1	K08907	3.102	1.712E-18	light-harvesting complex I chlorophyll a/b binding protein 1
*Lhca*2	K08908	2.435726227	7.151E-12	light-harvesting complex I chlorophyll a/b binding protein 2
*Lhca*3	K08909	2.773	1.233E-15	light-harvesting complex I chlorophyll a/b binding protein 3
*Lhca*4	K08910	2.194	0.01172	light-harvesting complex I chlorophyll a/b binding protein 4
*Lhca*4	K08910	4.519	1.454E-30	light-harvesting complex I chlorophyll a/b binding protein 4
*Lhca*5	K08911	3.747	2.208E-24	light-harvesting complex I chlorophyll a/b binding protein 5
*Lhcb*1	K08912	3.429	2.076E-21	light-harvesting complex II chlorophyll a/b binding protein 1
*Lhcb*2	K08913	3.949	4.592E-25	light-harvesting complex II chlorophyll a/b binding protein 2
*Lhcb*4	K08915	2.108	1.883E-09	light-harvesting complex II chlorophyll a/b binding protein 4
*Lhcb*5	K08916	2.472	8.85375E-13	light-harvesting complex II chlorophyll a/b binding protein 5

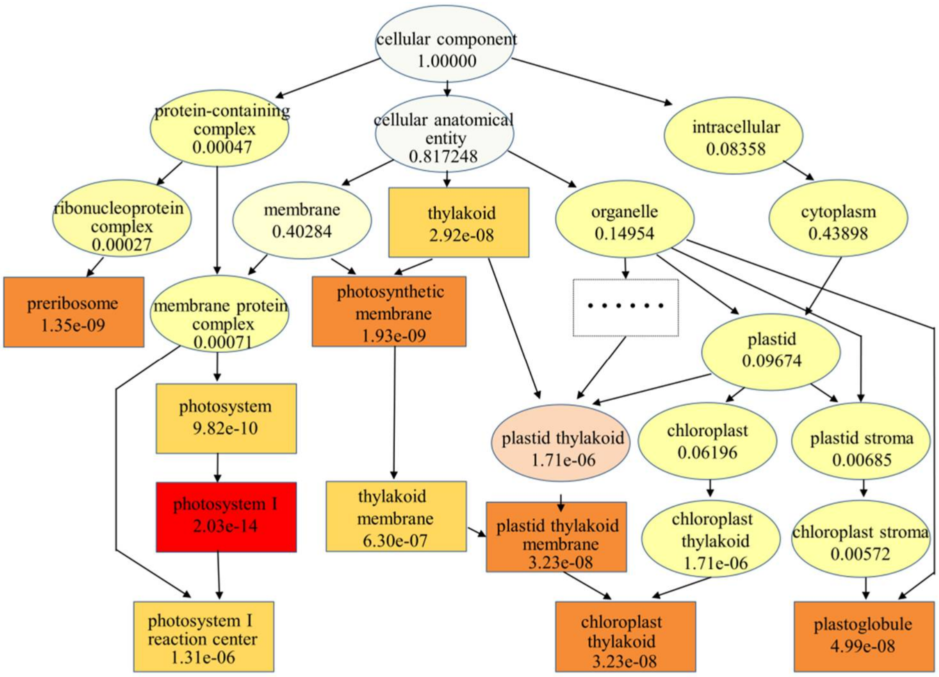

(A)

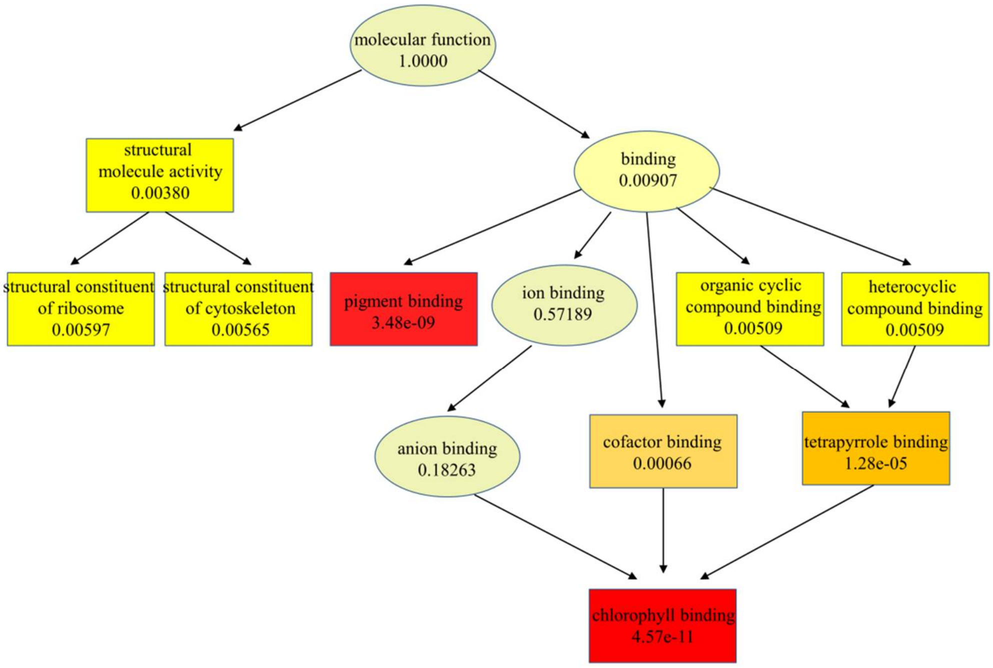

(B)

Fig. S1 Cytoscape visualization of the BiNGO clustering results of significantly enriched Gene Ontology (GO) cellular component (A) and molecular function (B) terms of up-regulated genes in *Scenedesmus obliquus* exposed to TC, compared to the control. The color of the nodes denotes the *p*-value (decrease with the deepening of

color from light yellow to deep red). The numbers in each term represent differentially expressed genes (DEGs) and total genes involved in this term. The top 10 GO terms are displayed in rectangular frames.
